# Glycothermal synthesis and photoluminescence of Mg–Si modified Ce:YAG nanophosphors†

**DOI:** 10.1039/d1na00060h

**Published:** 2021-03-31

**Authors:** Samuel Peter, Maureen Fitzpatrick, Adrian Kitai

**Affiliations:** McMaster University Department of Engineering Physics Hamilton ON Canada peters5@mcmaster.ca; McMaster University Analytical X-Ray Diffraction Facility Hamilton ON Canada; McMaster University Department of Materials Science and Engineering Hamilton ON Canada

## Abstract

The absorption spectrum of Ce in a YAG based host grown using the glycothermal method was modified using the addition of Mg–Si pairs. Photoluminescence intensity was dramatically improved by increasing the reaction temperature to 315 °C instead of the conventionally used 300 °C. It was found that Mg acetate and tetraethylorthosilicate (TEOS) are suitable as precursors for the glycothermal process, as EDS elemental mapping showed their homogeneous inclusion in the final product. Their addition only slightly modified the emission spectrum of Ce:YAG. It was found that increasing the reaction temperature to 315 °C yielded nanoparticles 56 ± 16 nm in size with a 3.3× enhancement in absorption and 3.7× enhancement in emission intensities compared to samples synthesized at 300 °C, and an increase in photoluminescence quantum yield from 32% to 48%. Reaction kinetics of the precursors and a proposed route for post-synthesis surface functionalization are discussed.

## Abstract 2

Influence of Mg–Si addition on absorption and emission of Ce_0.03_:Y_2.97_Mg_*x*_Al_5−2*x*_Si_*x*_O_12_ nanoparticles made using the glycothermal method.

## Graphical abstract

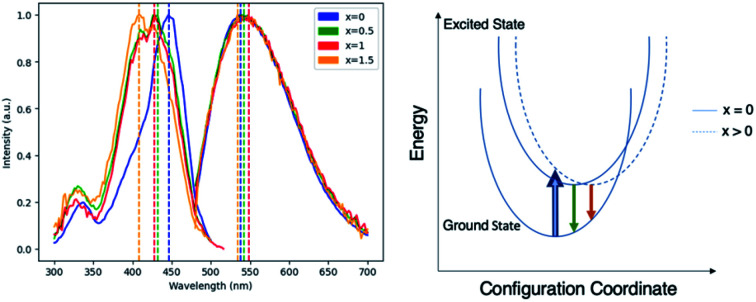

## Introduction

Oxidative degradation and lack of stability of OLEDs and QD materials and devices continues to be an issue for their long term use, especially in high brightness applications.^1–3^ Oxide phosphor materials continue to be important fluorescent materials in more robust inorganic LED devices. This article presents an alternative material system allowing for spectral tuning in well-known cerium-doped yttrium aluminum garnet nanophosphors (Ce:YAG).

Ce:YAG has been the workhorse of the solid state light community for the past several decades, as its absorption in blue and broadband yellow–green emission pair quite well with blue emitting InGaN LEDs to produce white light.^4–6^ In addition, as an oxide material, it is relatively immune to oxidative degradation, and is both thermally stable and chemically inert. YAG, or Y_3_Al_5_O_12_, as a host material crystallizes in the *Iā*3*d* space group, consisting of the yttrium atoms dodecahedrally coordinated to 8 oxygen atoms, and 5 aluminum atoms, 3 of which are tetrahedrally coordinated to 4 oxygen atoms, leaving the remaining 2 Al atoms octahedrally coordinated to 6 oxygen atoms.^7^ The Y site can accommodate large rare earth dopants such as Ce, which act as the luminescent centers in the YAG host.^8^ Luminescence of Ce originates from the allowed 5d → 4f transition, and as such, is not shielded by outer shell electrons and is therefore highly dependent on the local crystal field.^9^ Ce in the Y site of YAG experiences a strong ligand field imposed by 8 electron rich O atoms, which splits the degeneracy of the 5d excited state orbital to the point where electronic transitions are achievable with photons in the visible range.^9^

Ce:YAG has several drawbacks which limit or prevent its use in next generation lighting and display applications, mainly due to its particle size and its lack of a red component in the emission profile. The most common synthesis route involves repeated grinding and firing of Ce, Y, and Al oxides at temperatures above 1400 °C.^10–12^ These temperatures inevitably promote sintering and grain growth, leading to powders in the 10 μm size range. In microLED display applications, for example, the size of the phosphor powder is comparable to that of the subpixel, which can introduce optical crosstalk and excessive luminance variation between pixels and therefore a reduction in image sharpness and quality. A nano-sized Ce:YAG phosphor is more compatible with such microLED displays, and has the added advantage of reducing optical scattering. The optical scattering of ambient light limits the contrast ratio of the display.

Producing nano-sized Ce:YAG phosphors has been demonstrated by solution based synthesis approaches, such as the sol–gel method, co-precipitation method, ethanol–water solvothermal method, and glycothermal method.^13–21^ However, the sol–gel and co-precipitation methods require calcination at elevated temperatures, whereas the solvothermal and glycothermal approaches provide crystallized YAG directly from solution with no further annealing required. Further, the hydrothermal method demands high pressures and long reaction times in order to produce phase-pure YAG. Alternatively, the glycothermal method offers comparatively more moderate reaction temperatures, pressures, and reaction times to achieve phase pure, monodispersed YAG nanophosphors. The glycothermal method entails reacting metal alkoxide precursors in a high boiling point glycol solvent at elevated temperatures in a sealed reaction chamber. As a consequence of the metal alkoxide precursors used, organic species are typically observed on the particle surface upon completion of the synthesis.^23,24^

The lack of a red component in the emission profile of Ce:YAG has been an ongoing topic of research.^25–28^ Since the absorption and emission characteristics of Ce are directly dependent on the properties of the host, the usual approach for tuning Ce emission and absorption spectra involves modifying parameters of the local crystal field imposed by the host crystal.^29,30^ Gadolinium is often the dopant of choice, as it is larger in size than Y. Gd is optically inactive and it carries the same charge as Y.^26,31^ The larger ionic size of Gd distorts the dodecahedron outwards, which then shifts oxygen atoms closer to neighbouring Ce atoms, redshifting its emission. A similar strategy involves the use of Mg^2+^–Si^4+^ pairs in place of Al^3+^–Al^3+^ pairs in octahedral–tetrahedral co-ordination, respectively.^32–34^ In this case, a pair substitution is necessary to maintain overall charge neutrality.

The adoption of Ce into YAG *via* the glycothermal method has been studied extensively in recent years.^19–24,30^ However, most of the focus has been on an absolute size reduction of the nanophosphor, as opposed to tuning the optical properties. This study aims to demonstrate the ability to adjust the spectral properties of Ce in a YAG-based host with an *in situ* adoption of Mg^2+^–Si^4+^ pairs using the glycothermal method, not previously reported on to the best of the authors' knowledge.

## Experimental

### Synthesis of Ce_0.03_:Y_2.97_Mg_x_Al_(5−2x)_Si_x_O_12_ nanophosphors

Ce_0.03_:Y_2.97_Mg_*x*_Al_(5−2*x*)_Si_*x*_O_12_ nanophosphors were synthesized using the glycothermal method. Stoichiometric amounts of Y acetate hydrate (99.9%, Sigma Aldrich), Ce acetate hydrate (99.9%, Sigma Aldrich), Al isopropoxide (≥98%, Sigma Aldrich), Mg acetate hydrate (≥99%, Sigma Aldrich) were ground in an agate mortar before being added to 20 mL of 1,4 butanediol (1,4 BD, 99%, Sigma Aldrich) solvent with stoichiometric additions of tetraethylorthosilicate (TEOS, 98%, Sigma Aldrich) for Mg and Si values of *x* = 0, 0.5, 1, and 1.5. The mixture was magnetically stirred for 15 minutes before being added to a test tube held within a 53 mL autoclave fabricated using Swagelok Co. components. 8 mL of 1,4 BD solvent was held between the test tube liner and autoclave wall. The autoclave was purged with N_2_, sealed, and heated to 315 °C at a rate of ∼3.2 °C min^−1^ over 90 minutes. The samples remained at 315 °C for 3 hours, with stirring at 300 rpm *via* a magnetic stir bar on a hot plate. Once cooled to room temperature, the pale yellow nanophosphors were washed three times by centrifugation at 15 000 rpm with ethanol, and dried overnight at 90 °C. The same procedure was repeated for samples made with *x* = 0 at 300 °C.

### Characterization

The crystal structure of the YAG based nanophosphors was confirmed using X-ray diffraction. A Bruker D8 DISCOVER diffractometer with Co Kα source (*λ*_avg_ = 1.79026 Å) was used, and diffraction peaks were referenced to Y_3_Al_5_O_12_ (ICDD PDF #00-033-0040). Rietveld refinement was performed in the TOPAS software to determine microstructure parameters. Electron microscopy was performed on a Thermo Scientific TALOS 200X transmission electron microscope followed by diameter measurements using National Institute of Health (NIH) ImageJ software. Confirmation of Mg and Si inclusion was achieved using EDS mapping on the same TEM operating in STEM mode with a high angle annular dark field (HAADF) and four in-column SDD Super-X detectors. Thermogravimetric analysis was performed on the precursor materials under 30 mL min^−1^ Ar flow using a Mettler Toledo TGA-DSC 3 + system in the range of 25 °C to 600 °C by heating 25 °C to 100 °C at a rate of 20 K min^−1^, followed by a 5 minutes isothermal stage at 100 °C to expel any residual moisture, before proceeding to the temperature range of 100 °C to 600 °C at a rate of 10 K min^−1^. Photoluminescence (PL) and photoluminescence excitation (PLE) measurements of the formed nanophosphors were performed on a Tecan Infinite M200 Pro Plate Reader by making solutions of dried nanoparticles in ethanol at a concentration of 5 mg mL^−1^ with dispersion *via* ultrasonication for 10 minutes. PL/PLE measurements for samples synthesized at 300 °C and 315 °C were obtained at fixed concentrations of 200 μg mL^−1^ to reduce the effect of optical scattering. Photoluminescence quantum yield (PLQY) of powder samples synthesized at 300 °C and 315 °C were evaluated using an integrating sphere (LabSphere) and spectrometer equipped with a CCD detector (Ocean Optics 2000+) with a 375 nm diode laser (Coherent OBIS LX) acting as the excitation source. Full details of this measurement can be found in the ESI.†

### Surface modification of nanophosphors

Surface modification of the formed Ce:YAG nanophosphors was carried out by washing the nanoparticles with centrifugation in a 0.1 M HCl solution three times, followed by washing in distilled water three times before dispersing in 20 mL distilled water. Citric acid was added in a 1 : 2 weight ratio between citric acid and the nanopowder, and the mixture was stirred at 700 rpm at 75 °C for 30 minutes on a hot plate. The product was then washed three times by centrifugation with distilled water and dried at 120 °C overnight. The presence of surface organic groups was verified after nanoparticle synthesis, washing with HCl, and after addition of citric acid using a Bruker Hyperion 3000 FTIR spectrometer operating in attenuated total reflectance (ATR) mode in a range from 4000–400 cm^−1^.

## Results and discussion

### Structural and optical characterization of Ce_0.03_:Y_2.97_Mg_x_Al_(5−2x)_Si_x_O_12_ nanophosphors

**Fig. 1:**
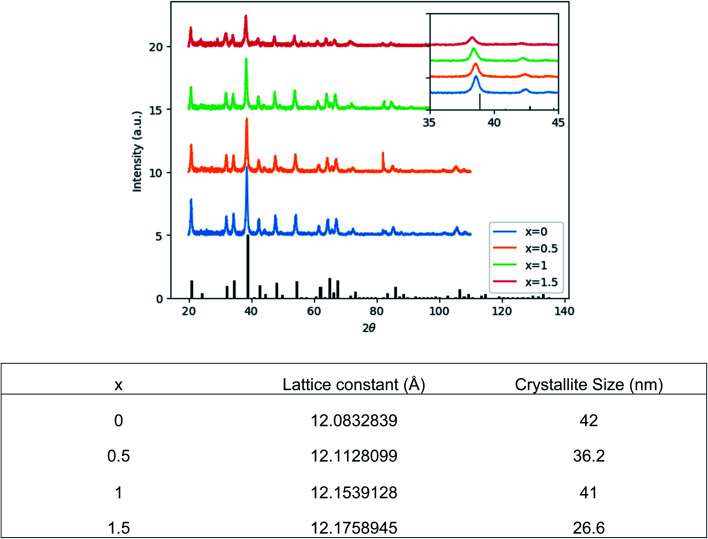
(Top) XRD spectra of dried Ce_0.03_:Y_2.97_Mg_*x*_Al_(5−2*x*)_Si_*x*_O_12_ powders, with *x* = 0, 0.5, 1, 1.5. Referenced to ICDD PDF# 00-033-0040. Inset: magnified view of 38.5° 2*θ* peak. (Bottom) Microstructure parameters obtained by Rietveld refinement.

**Fig. 2:**
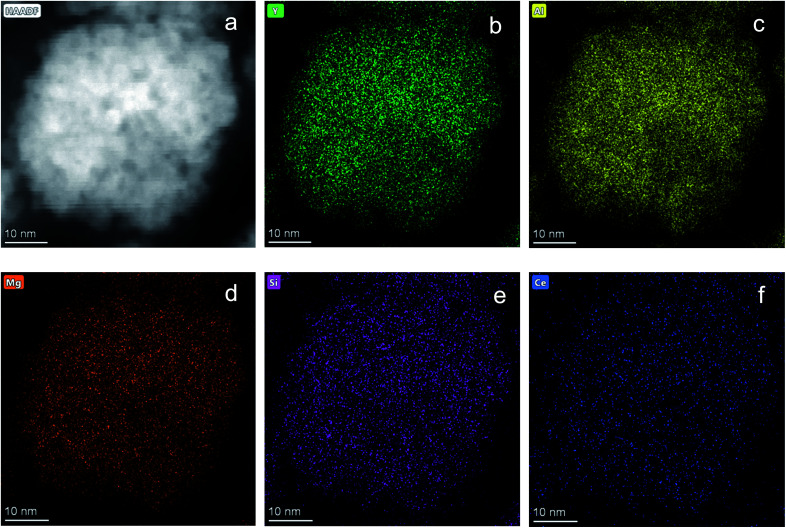
EDS mapping of Ce_0.03_:Y_2.97_Mg_*x*_Al_(5−2*x*)_Si_*x*_O_12_ with *x* = 1. (a) STEM image of area mapped. Elemental mapping of (b) Y, (c) Al, (d) Mg, (e) Si, (f) Ce. Scale bar indicates 10 nm.

Fig. 1 shows the structural evolution of the formed Ce_0.03_:Y_2.97_Mg_*x*_Al_(5−2*x*)_Si_*x*_O_12_ nanopowders using the glycothemal method, with *x* values of 0, 0.5, 1, 1.5. The obtained spectra are referenced to that of pure YAG (ICDD PDF# 00-033-0040). The inset of Fig. 1 highlights the shift in the dominant 2*θ* peak around 38.5°, likely caused by larger Mg ion substitution at the Al octahedral site. This is thought to be the case due to the similar size of the Mg^2+^ ion (78 pm) and the Al^3+^ ion at the octahedral site (68 pm), and Si^4+^ (39 pm) substituting for Al^3+^ at the tetrahedral site (53 pm).^33^ Results of the Rietveld refinement, summarized in Fig. 1, show that the lattice spacing increases fairly linearly with increasing Mg–Si addition. In order to confirm the inclusion of Mg and Si in the final product, EDS mapping was undertaken, shown in Fig. 2. Fig. 2 shows a uniform distribution of Mg and Si, indicating a homogeneous inclusion during the glycothermal reaction.

**Fig. 3:**
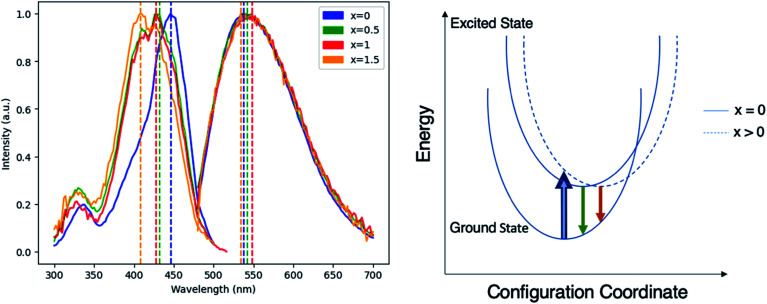
(Left) Normalized PLE and PL spectra of Ce_0.03_:Y_2.97_Mg_*x*_Al_(5−2*x*)_Si_*x*_O_12_, with *x* = 0, 0.5, 1, and 1.5. Dashed vertical lines indicate spectral maxima for PLE and PL, respectively: 446 nm and 538 nm for *x* = 0, 432 nm and 542 nm for *x* = 0.5, 428 nm and 548 nm for *x* = 1, and 408 nm and 534 nm for *x* = 1.5. (Right) Schematic of Franck–Condon principle demonstrating the origin of absorption blueshift and emission redshift in Ce_0.03_:Y_2.97_Mg_*x*_Al_(5−2*x*)_Si_*x*_O_12_.

To evaluate the viability of Mg–Si addition to alter the optical properties of Ce:YAG, photoluminescent excitation (PLE) spectra and photoluminescent emission (PL) spectra were obtained, as shown in Fig. 3. The general trend was that with the increased addition of Mg and Si, there was a blueshift in the absorption, and a small redshift in the emission. This is likely attributed to the Franck–Condon principle, which describes how a spatial offset between the ground and excited state, such as that induced by the Mg–Si addition, can alter the energy of the absorption and emission transitions.^35^ Moreover, it describes the intensities of the observed transitions as an indication of the degree of overlap between the ground and excited state wavefunctions. The spectra presented in Fig. 3 are normalized in order to highlight shifts in the spectra, however it was observed that the intensity of absorption and emission decreased as the Mg–Si content increased. This may also explain the discrepancy in the *x* = 1.5 emission profile, as the signal-to-noise ratio in this case was low. With Mg^2+^–Si^4+^ substitution, the effect on Ce redshifting can be two-fold: the larger ionic size of Mg^2+^ compared to Al^3+^ distorts the octahedron outwards, and the oxygen atoms in the Si–O bond experience an increased degree of covalency compared to that of Al–O, which are then stabilized with an outward coulombic repulsion.

### Effect of increased reaction temperature on Ce:YAG nanophosphors

**Fig. 4:**
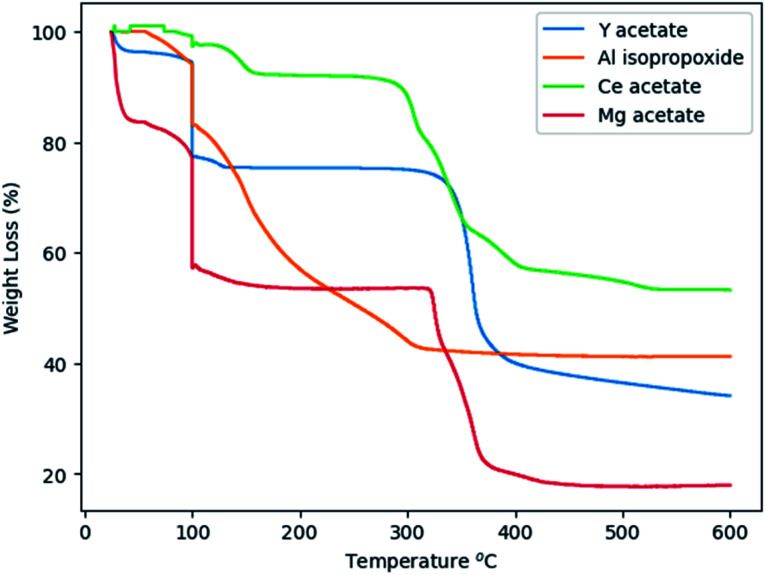
TGA spectra of precursor alkoxide powders.

Glycothermal synthesis of Ce:YAG was first presented in 2006 by Isobe *et al.* by reacting yttrium and cerium acetates with aluminum isopropoxide in 1,4 butanediol, and the reaction temperature to produce phase pure YAG was 300 °C. Since then, it has generally been accepted that Ce:YAG nanophosphors are to be synthesized at 300 °C.^21–23^ However, there has been little study into the behavior of the precursor materials at those temperatures in the context of reaction kinetics. As such, thermogravimetric analysis (TGA) was performed on the precursor powders to get an indication of at what temperatures the C–O bond of the metal–organic precursors cleave *via* thermal decomposition, as shown in Fig. 4. There is an initial significant weight loss at 100 °C, which is attributed to vaporization of residual moisture. It was observed that the majority of weight loss for Y, Ce, and Mg precursors occurred above 300 °C, although the initial bond cleavage for Al occurred well below this temperature.

**Fig. 5:**
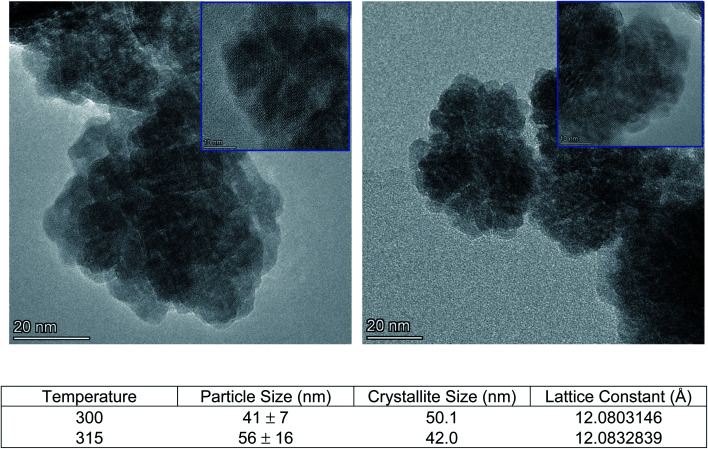
TEM images of formed Ce:YAG at 300 °C (left) and 315 °C (right). Inset: HRTEM images (scale bar is 10 nm). (Bottom) Summary of microstructure between samples made at 300 °C and 315 °C. Particle size measured from TEM images, crystallite size and lattice constant calculated using Rietveld refinement of XRD data.

**Fig. 6:**
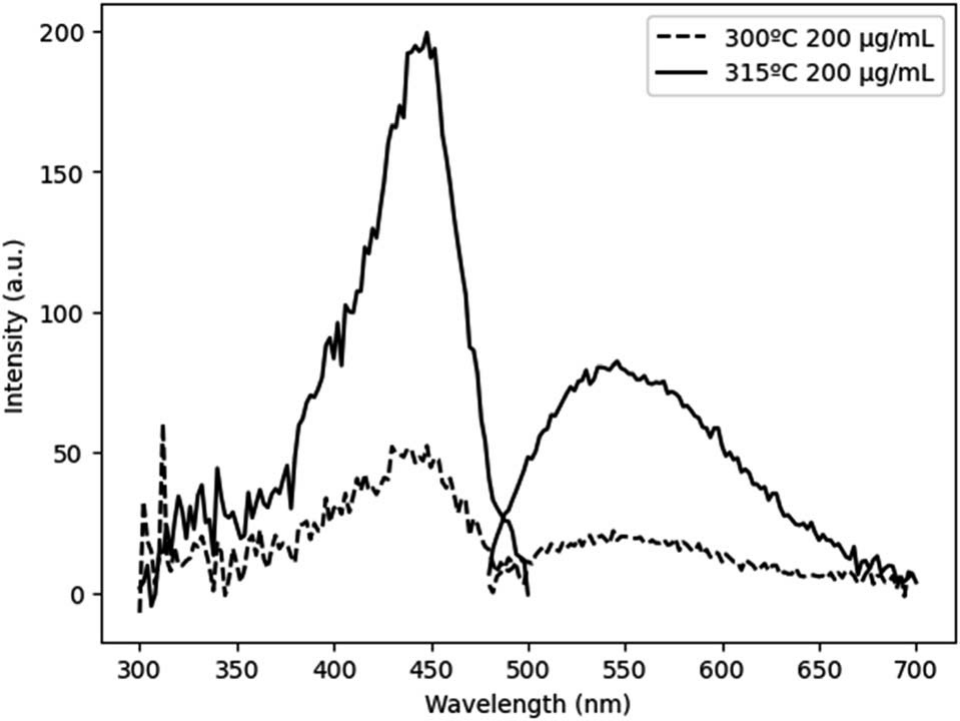
PLE and PL of Ce:YAG with for 300 °C (dashed line) and 315 °C (solid line). Samples dispersed in ethanol at a concentration of 200 μg mL^−1^. PLQY values of samples made at 300 °C and 315 °C were 32% and 48%, respectively.

Cleavage of the C–O bond provides a site for nucleation to occur, and it is therefore critical that all precursors are available for reaction at a similar time in order to facilitate homogeneous incorporation in the final product. This approach comes at the cost of particle size and size distribution control, as an increased reaction temperature encourages higher precursor dissolution and therefore precursor availability during the growth phase.^36^ This can be seen in Fig. 5, comparing Ce:YAG synthesized at 315 °C *vs.* 300 °C. The average particle sizes measured from TEM images were 56 ± 16 nm, and 41 ± 7 nm, respectively, in reasonable agreement with the 42.0 nm and 50.1 nm for 315 °C *vs.* 300 °C samples calculated from Rietveld refinement. The optical performance of the nanophosphors at 315 °C *vs.* 300 °C was evaluated and is presented in Fig. 6. The higher temperature shows a marked improvement in optical performance, with a 3.3× enhancement in PLE and a 3.7× enhancement in PL. In addition, the photoluminescence quantum yield (PLQY) increased from 32% for samples made at 300 °C to 48% for those made at 315 °C. This may be due to an improvement in the homogeneity of the dodecahedrally coordinated Ce atom sites, which reduces the potential for non-radiative transitions *via* dopant clustering. In addition the increase in particle size results in a smaller surface area-to-volume ratio compared to that for the smaller particles synthesized at the lower temperature. This reduces the influence of surface defects. Surface defects and surface states associated with dangling bonds are well known to quench luminescence.^24,37^

### Surface modification of Ce:YAG nanoparticles

**Fig. 7:**
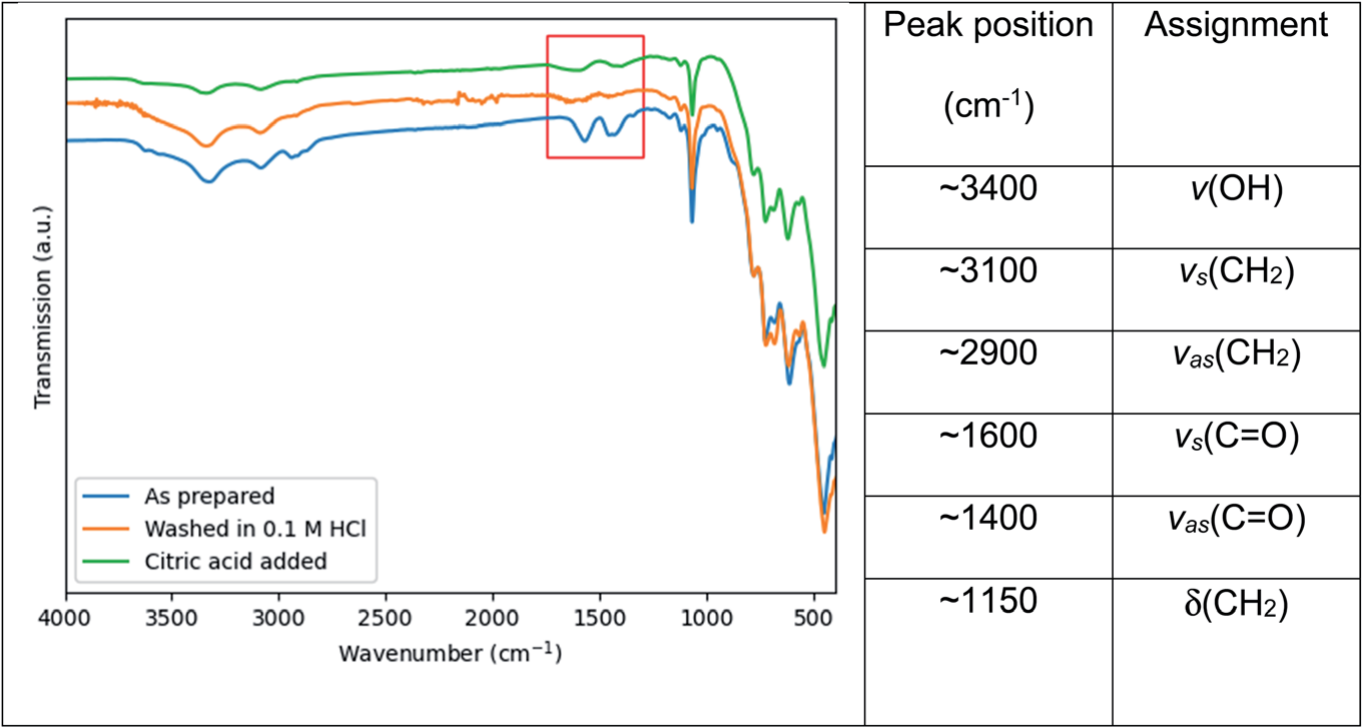
FTIR spectra of as prepared Ce:YAG samples after stripping of surface organic groups with a 0.1 M HCl solution, and after grafting with citric acid. The region of interest, namely the symmetric and asymmetric stretching of C=O bonds at ∼1600 cm^−1^ and ∼1500 cm^−1^ respectively, is highlighted in red. Peak assignments are taken from ref. 23 and ref. 38.

In an attempt to broaden the range of functionality of the Ce:YAG nanoparticles for integration in a wider variety of applications, the residual organic groups on the particle surface were removed and replaced with citric acid, as shown by the surface sensitive FTIR-ATR results in Fig. 7. The peaks highlighted in red of Fig. 7 are attributed to the symmetric and asymmetric stretching of C=O bonds at ∼1600 and ∼1500 cm^−1^ respectively, and originate from residual acetate groups from the Y and Ce precursors remaining on the particle surface, which are removed after washing in a mild acidic solution. Upon heating in a solution containing citric acid, the peaks appear again, indicating the re-formation of the C=O bond and therefore the grafting of citric acid on the particle surface. This relatively simple and straightforward technique can potentially improve the compatibility of Ce:YAG nanoparticles with epoxies, polymers, or composites provided an appropriate C=O functional group anchor can be formed.

## Conclusion

The optical properties of Ce:YAG were modified by the addition of Mg–Si pairs to the host lattice using the glycothermal method. A moderate blueshift in absorption and slight redshift in emission was observed. Increasing the reaction temperature by 15 °C provided a significant improvement in both absorption and emission. This increases the versatility of Ce:YAG – based nanophosphors permitting, for example, the nanophosphor absorption spectrum to be better tuned to the emission spectrum of a blue microLED source emitting between 400 and 450 nm. A technique is proposed to further modify the nanoparticle surface post-synthesis which can, in future, enable the nanophosphor to be incorporated in organic binders.

## Conflicts of interest

There are no conflicts to declare.

## Supplementary Material

NA-003-D1NA00060H-s001
