# Supplementary material for: Glycothermal synthesis and photoluminescence of Mg–Si modified Ce:YAG nanophosphors
Source: Nanoscale Adv. 2021 Mar 31;3(10):2911–7. doi: 10.1039/d1na00060h (PMC9417575; doi:10.1039/d1na00060h)
Supplement: NA-003-D1NA00060H-s001 [file NA-003-D1NA00060H-s001.pdf]

Electronic Supplementary Material (ESI) for Nanoscale Advances.
This journal is © The Royal Society of Chemistry 2021

Glycothermal synthesis and photoluminescence of Mg-Si modified Ce:YAG nanophosphors

Supplementary Information

Calculation of Photoluminescence Quantum Yield

Photoluminescence quantum yield (PLQY), or internal quantum efficiency, of dried nanopowders synthesized at 300ºC and 315ºC was calculated using:

$$PLQY = \frac{\phi_{emitted}}{\phi_{absorbed}} \tag{1}$$

where $\phi_{emitted}$ and $\phi_{absorbed}$ is the flux of the emitted and absorbed photons in s^{-1}, respectively. Photon fluxes were calculated using a 3.3" Spectralon coated integrating sphere (LabSphere) connected to a spectrometer with CCD detector (Ocean Optics 2000+) via a 600 µm diameter visible-NIR fibre optic cable with a numerical aperture of 0.22, and a 375 nm diode laser (Coherent OBIS LX) acting as the excitation source. The integrating sphere contained two 0.5" diameter ports for the laser and fibre optic connection, respectively. A calibrated tungsten halide light source (LS-1-CAL, Ocean Optics) was used to convert detected counts into a spectral irradiance $I(\lambda)$. An integration time of 100 ms was used.

The response of the integrating sphere, a unitless quantity which accounts for the reflectivity of the sphere coating, coupling to the fibre optic cable, and light lost through the sphere ports was calculated using:

$$R_s(\lambda) = \frac{A_{fibre} \cdot (1 - R) \cdot \Omega \cdot \rho}{\pi A_{sphere} \cdot [1 - \rho(1 - f)]} \tag{2}$$

Where $R_s(\lambda)$ is the sphere response, A_{fibre} is the cross-sectional area of the fibre, R is the reflectivity of the fibre face, Ω is the solid angle subtended by the fibre (approximated as $\pi \cdot (numerical\ aperture)^2$), ρ is the reflectivity of the sphere coating, A_{sphere} is the surface area of the sphere, and f is the port factor, or the ratio of areas of the ports to the sphere surface area. The sphere reflectivity was then fitted to a third order polynomial function.

Photon fluxes were then determined using:

$$\phi_{incident} = \int_0^\infty \frac{I_{laser}(\lambda) \cdot R_s(\lambda)^{-1}}{hc/\lambda} \cdot d\lambda \tag{3}$$

$$\phi_{reference} = \int_0^\infty \frac{I_{reference}(\lambda) \cdot R_s(\lambda)^{-1}}{hc/\lambda} \cdot d\lambda \tag{4}$$

$$\phi_{emitted} = \int_{390\ nm}^{710\ nm} \frac{I_{sample}(\lambda) \cdot R_s(\lambda)^{-1}}{hc/\lambda} \cdot d\lambda \tag{5}$$

$$\phi_{sample} = \int_{380\ nm}^{360\ nm} \frac{I_{sample}(\lambda) \cdot R_s(\lambda)^{-1}}{hc/\lambda} \cdot d\lambda \tag{6}$$

Where $\phi_{incident}$, $\phi_{reference}$, $\phi_{emitted}$, and ϕ_{sample} are the fluxes of the laser with no sample in place, the sample holder (a low density polyethylene bag), sample emission, and scattering of the sample in the laser beam respectively. The flux of photons absorbed by the sample was determined as:

$$\phi_{absorbed} = \phi_{reference} - \phi_{sample} \tag{7}$$

PLQY was then calculated according to equation (1).

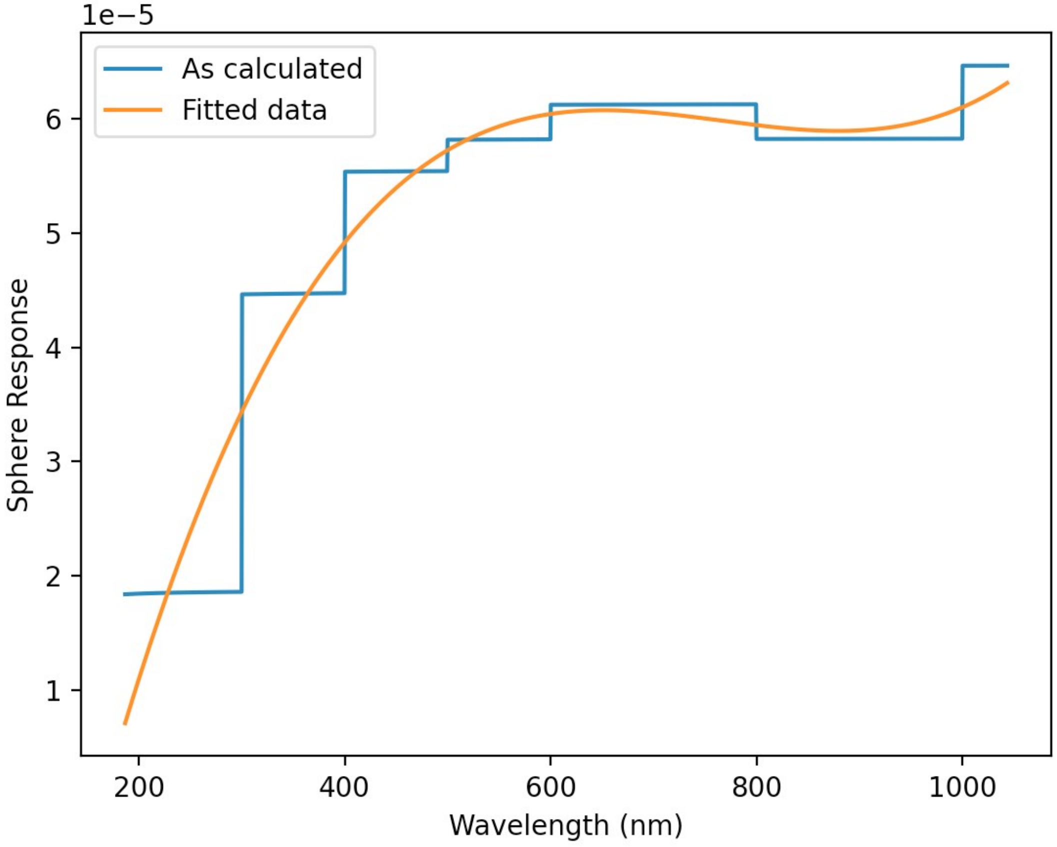

Figure S1: Sphere response with fitted curve. Data fit to $f(x) = ax^3 + bx^2 + cx + d$ *with [a, b, c, d] = [3.07721257e-13, -7.06784320e-10, 5.29177847e-07, -6.90925893e-05]. Step function of sphere response was due to low resolution in sphere coating reflectivity.*
